# Foreign Summary

**Published:** 1840-06-06

**Authors:** 


### FOREIGN SUMMARY.

*On a remarkable effect upon the Gums produced by the slow introduction of lead oxide into the human body.* By Henry Burton, M. D., Physician of St. Thomas's Hospital.—In explanation of the circumstances by which the author was first induced to investigate the action of lead oxide on the gums, Dr. Burton says he had been taught to believe from the perusal of Dr. Warren's Essay on the Effects of Lead, published in 1772, and of Dr. Christison's description of the symptoms produced by the same oxide on man, published in 1829, and republished in 1836, that a salivation was occasionally excited by its slow introduction into the human body, and during which the saliva was increased in quantity, as well as rendered (according to Dr. Christison) bluish in colour.

In no other author, among several which were consulted, could Dr. Burton meet with any additional notice of unusual symptoms having reference to the state of the mouth produced by the absorption of lead oxide. But his attention was first practically directed to its influence on the salivary glands, in the year 1834, when his friend Dr. Roots, and late colleague at St. Thomas's Hospital, had a patient in one of his wards who was said to have been salivated by the internal use of plumbi acetas.

From that period to the present time, an interval of about five years, Dr. Burton has continued the examination of the mouths of patients who have been admitted into his ward with lead colic and lead paralysis; the result of which has been a belief that a salivation, in the ordinary sense of the word, does not occur in one case out of twenty-eight well marked cases of disease, from the absorption of lead oxide, which number have come under the treatment.

Dr. Burton does not deny that salivation has occurred and may occur again; but he contends that a peculiar appearance is invariably produced by lead oxide on the gums, and which may be considered as indicative of its presence in the system. On these twenty-eight patients the edges of the gums, where they were attached to the necks of two or more teeth of either jaw or both jaws, were distinctly bordered by a narrow line of a deep leaden blue colour, about the 1-20th part of an inch in width, whilst the remainder of the gums, for the most part, retained their usual colour and condition. This phenomenon, observed on the gums of patients affected by lead oxide, differs entirely from any one characteristic of the presence of mercury in the system, as well as of scorbutus; and is never seen unless the patient has been exposed to the long-continued operation of lead oxide.

In support of these opinions Dr. Burton adds, that he has intentionally produced the peculiar appearance alluded to by the internal use of plumbi acetas, and that he was unable to distinguish it on the gums of fifty-two hospital patients under treatment for various diseases, which were not complicated with either lead colic or lead paralysis. He is, therefore, inclined to rely on this symptom as an infallible proof of the presence of lead oxide in the system; and, that in all cases of illness originating from this oxide, about the symptoms of which some ambiguity exists, an examination of the gums will materially assist in making a correct diagnosis.

The author notices briefly, the conditions of disease in which this ambiguity is sometimes remarked, and asserts that, in the majority of cases of which lead oxide is the cause of illness, a careful inspection of the gums will immediately reveal the origin of the evil, and suggest an appropriate plan of treatment.

In six cases in which plumbi acetas was administered internally by Dr. Burton, the appearance of the narrow leaden blue border line preceded the accession of other symptoms indicating the presence of lead oxide in the system; and the use of the salt was in consequence discontinued. In two of these cases colic symptoms followed its appearance, but in the remaining four they did not follow. This sign, therefore, Dr. Burton thinks cannot be implicitly relied on as a means of always averting the pains of lead colic; nevertheless, he believes it may be depended on with as much safety as the copperish taste of the saliva is confided in as an indication to withhold the farther use of calomel, for the purpose of avoiding the other symptoms of mercurial salivation.

During the discussion which ensued at the meeting of the Society after Dr. Burton's paper had been read, Dr. George Burrows stated that a corresponding colouration of the gums had been observed in Germany, and an account published of it in Fromp's Notizen, No. 246, for October, 1839.

We have been since favoured with a translation of that account, from which it appears that the author (Dr. Schilbach, of Neustadt,) in the month of August, 1839, was called into a consultation upon the health of a father and five children who had been poisoned by the use of bread containing lead oxide. On all the patients (Dr. Schilbach observed) an almost characteristic ash gray coating of the gums "at the part where they surrounded the teeth." No farther notice is taken of this appearance by Dr. Schilbach. The sentence above quoted, however, strongly confirms the accuracy of Dr. Burton's observations, whilst it in no way deprives him of the priority of discovery; and we have authority for unequivocally denying on his part any knowledge of Dr. Schilbach's paper previous to the discussion which took place at the Medical and Chirurgical Society.—*Lond. Med. Gaz.*

*Aneurism of the Ascending Aorta.* By M. Dubois.—At the meeting of the Royal Academy of Medicine of Paris, 5th December, 1839, M. Dubois exhibited an interesting specimen of morbid anatomy, illustrative of the tendency of aneurismal as well as other tumours, to approach the surface, notwithstanding every natural obstacle. The diseased vessel was the ascending aorta, from which, while yet within the pericardium, might be seen a tubular prolongation, of the diameter of two inches, proceeding at right angles directly outwards, and divided near its middle in two unequal parts, by a kind of circular valve. Externally, this tube communicated with a large pouch of condensed cellular tissue, lying beneath the pectoral muscles, and extending into the axilla. Three intercostal spaces had been perforated, not only the intercostal muscles, but one of the ribs having been absorbed as the tumour grew, so that the rough edges of the bone were in contact with the fibrous coagula of the aneurism.

The whistling respiration and œdema of the upper extremities observed during life clearly arose from the pressure of the tumour.

[The foregoing case confirms the truth of the observation that aneurisms of the arch of the aorta more commonly rise towards the sternal surface of the chest. Corvisart, Duverney, Morgagni, and others, have given instances of this kind.]—*Brit. and For. Med. Rev., from Bulletin de l'Académie de Médecine.* December 31, 1839.

---

*On the Various Circumstances which appear, in the Course of Diseases, to produce the Curved Form of the Nails.* By M. Vernois.—This paper is sufficient to determine the value, or rather the want of value, of curved finger-nails as a sign of disease. The results obtained from the examination, by the numerical method, of two hundred and seventy-six cases of various diseases, are as follows:

In any collection of patients, whatever be their diseases, curved nails will be found in at least one-third. Among different diseases, phthisis, scrofula, and other chronic affections, have a very marked, though not an absolute or constant influence. Of eighty-eight patients with curved nails, seventy were phthisical or scrofulous; and of one hundred and eighty-eight with normally formed nails, forty were suffering from the same conditions. Of the same eighty-eight, nine only laboured under acute diseases. Females present this alteration about three times more frequently than males. It is most commonly observed between the ages of ten and thirty. Occupation has no influence upon it. The constitution which coincides with it in five sixths of the cases in which it occurs is that which is marked by a pale, fine, and ænemic skin, blond hair, blue or brown eyes, bluish sclerotica, very long eyelashes, and weak muscles.—*Ib.*,

*from Archives Générales de Médecine.* November, 1839.

*On the Use of Hydrochlorate of Baryta in Strumous Ophthalmia.* By Dr. PAYAN, of Aix in Provence.—Dr. P. having observed that this remedy was used with excellent effect by Lisfranc in scrofulous diseases, he resolved to try it in an obstinate case of ophthalmia accompanied by a high degree of photophobia. The patient was six years of age. He dissolved two grains of the hydrochlorate in three ounces and a half of "eau sucrée," and this quantity was taken in portions during the course of the day. No particular effect being produced, on the third day three grains were taken, and the dose was gradually raised to ten grains in the course of the day. On the twentieth day the medicine was discontinued, the patient being considered nearly well. In another case, twelve grains in the course of the day were taken with excellent effect, and without any symptoms of gastric irritation being produced. During the administration of the remedy, Dr. Payan orders a light and sparing diet, considering that harm is frequently done by the tonic medicines and stimulating regimen which are generally ordered as a matter of course in strumous ophthalmia. It is true that the scrofulous constitution is often accompanied by an atony which is marked by a pale and pasty complexion, feeble circulation, a low degree of sensibility, and a general indolence of mind and body; but the same scrofulous disposition is frequently developed in persons of a lively temperament, animated countenance, habitually quickened circulation, and mobile and easily excitable nervous system. It is in this latter class of persons that we most frequently find that acute sensibility of the retina which produces photophobia, with the accompanying spasmodic contraction of the eyelids, and abundant secretion of tears. In these cases, a generous diet, with wine and tonics, will increase the general excitement, and consequent irritability of the retina, whilst the barium appears to act as a sedative, and, combined with a mild diet, produces a general soothing effect.—*Ib., from Revue Médicale.* April, 1839.

*On the Treatment of Dysentery with Albumen.* By M. SAUCEROTTE, Physician to the Hospital at Lunéville.—The dysentery, which annually attacks a considerable number of men of our garrison, affected only thirteen in the months of October and November, 1838, and ten in those of August and September, 1839. I employed in some of these cases the albuminous method of treatment recommended by MM. Bodin de la Pichonnerie and Mondière. Comparatively with those by the ordinary method, (by antiphlogistics and opiates,) the results obtained by it were really of the most striking kind. My own experience, and that acquired by others in the hospital, shows that when the disease is at all severe, it generally lasts two or three weeks, sometimes becomes chronic, or terminates in death: besides this, the reiterated application of leeches causes severe pain, the administration of enemata is continually attended with much difficulty, the convalescence is prolonged, and attended with extreme weakness when the disease is not stopped during the first week. Judging from the cases I have observed, the albuminous treatment is attended with precisely contrary effects—rapid cessation of the symptoms, quick convalescence, and recovery of strength, while the administration is attended neither with pain nor any sort of inconvenience.

[The albumen was exhibited both as a ptisan and in enemata, according to the formula of M. Mondière, and eight cases are related (some, however, with ludicrous brevity) in support of the above emphatic encomiums. In some of the cases there was undoubted inflammation of the large intestine; in all frequent bloody stools, tenesmus, &c.]—*Ib., from Gaz. Médicale de Paris.* No. xlvii. Nov., 1839.

*On the Treatment of Malignant Ulcers by Cauterization with Corrosive Sublimate.* By M. ORDINAIRE.—M. Ordinaire for the last fifteen years has been in the habit of employing the bichloride of mercury as a caustic in the treatment of cancerous, scrofulous, venereal, and other intractable ulcers, and has never observed the least symptom of absorption, which he ascribes to the instantaneousness of its destructive action on the absorbents, and to its being decomposed by the albumen of the tissues. The quantity of the powder employed varies with the thickness and the nature of the parts requiring to be destroyed, but never exceeds seven or eight grains. If the cancerous ulceration be superficial, a single application of caustic suffices; but it is generally necessary to repeat it after the separation of the eschar. He also employs this salt for the cauterization of strictures of the urethra. [The following, the third case related, is a specimen of the activity of this surgeon's practice:] M. I., innkeeper, subject from infancy to prolapsus of the gut, began at ætat twenty to experience much difficulty in reducing it after defecation, which increasing till his thirty-second year, reduction then became impossible. M. O., on examining the patient at this period, found at the orifice of the rectum a tumour as large as an egg, traversed by several deep sulci, dividing the mass into ulcerated lobes. The tumour was hardly tender to the touch, yet caused severe lancinating pain; it bled when handled, and discharged a very fœtid, icherous matter. Recognising cancerous degeneration, the author removed with a bistoury the mass protruding beyond the anus, and having ascertained that diseased excrescences extended two inches up the rectum, determined on removing them with the bichloride. He prepared a cylindrical plug of shredded lint of the thickness of the finger, and four or five inches long, smeared it with a glutinous matter, then sprinkled it all over with the caustic, and pushed it two inches and a half up the rectum. Severe pain soon came on, and there was great difficulty in persuading the patient to bear the plug for a few hours. Sloughs as large as an almond separated on the fourth day with the aid of injections and hip-baths, and further application of the caustic was only required to two spots at the margin of the anus, which had a suspicious appearance. The patient was entirely cured thirty-two days after the first cauterization; the sphincters performed their office well. The operation was performed twelve years since, and the patient enjoys perfect health.—*Ib.*, *from Gaz. Médicale de Paris.* No. xlvi. 1839.

---

*Cure of an Old Dislocation of the Humerus by Division of the Pectoralis Major, Latissimus Dorsi, Teres Major, and Teres Minor Muscles.* By Professor DIEFFENBACH.—Herr Th., a large landowner, upwards of thirty years old, had his right shoulder dislocated two years ago by a fall from his horse; the nature of the accident was not at first recognised, and afterwards, though all the usual means were adopted by several surgeons, the bone could not be returned to its place. The patient, therefore, came to Berlin; he was of a gaunt, powerful form, with a pale complexion and but little fat, and his muscles were strong and prominent under the skin. The injured right shoulder was an inch higher than the left; the acromion formed a short angle; on the outer side the shoulder was deeply hollowed, and the scapula lay flat. The right arm was thinner than the left, and stood out far from the body. The head of the humerus lay on the anterior side of the chest, close to the clavicle, and two inches from the upper portion of the sternum. The patient had a constant sensation of cold in the limb, and the creeping which he had formerly felt had ceased. The pulse in the right radial artery was rather weaker than that in the left. The limb was useless, and only the hand could perform some slight actions.

By moving the arm in different directions, severe pain was produced in the part where the head lay surrounded by a thick wall of dense ligament into which it had worked itself. In drawing the arm outwards from the body, the pectoralis major, latissimus dorsi, teres major, and teres minor, became tense with extreme pain. The last three of these muscles felt hard and tense, even when the arm was not drawn outwards. An attempt to reduce such a dislocation without dividing these muscles and the new joint would have been extremely dangerous, and had been found impossible; but (says the Professor) I anticipated success from the subcutaneous division of every thing that resisted me.

The patient being placed on the table, with one folded sheet passed under the right axilla, and held by six assistants, another fastened round the right hand and held by six more, and a third round the upper part of the humerus held by three more, (in the manner usually adopted by me in old luxations,) the two first sets of assistants were ordered to pull against each other. I next bade them make a slowly increased extension, and then stop; I then passed a small scythe-shaped knife through the skin, and divided the most tense portion of the pectoralis major close to its tendon, which yielded with a cracking sound. I then again introduced the knife at the posterior border of the axilla, and divided one after the other the latissimus dorsi, the teres major, and the teres minor. All these muscles gave way with a cracking noise, which was increased by the resonance of the chest. I next passed my knife into three places by the head of the humerus, and divided in a similar manner under the skin the dense and hard false ligaments which surrounded the new joint, and lessening the extension, I loosened the head by a few rotations.

A powerful extension was now again commenced on both sides, and the three assistants behind the patient pulled suddenly while I conducted the humerus towards the joint into which it slipped on a sudden, without again springing out. One shoulder looked now just like the other. The thorax, the shoulder, and the arm, were enveloped with bandages, which were soaked with paste, and after a few hours they all became dry and hard, and prevented any motion of the right side.

The bleeding from the wounds, which were not larger than those made in phlebotomy, was at most a few drops. No unpleasant symptoms ensued, and the patient suffered even less than the majority of persons in whom I have reduced old dislocations. On the ninth day I took off the bandage; both shoulders had exactly the same level and form, and there was neither swelling nor pain. The punctures in the axilla had completely healed, and scarcely a trace of them could be found; there was no collection of blood or pus. The arm was already capable of motion, and its actions were far less hindered than they are sometimes after the reduction of recent dislocations; because in them there is often for a long time a sensitive contraction of the unnaturally stretched muscles, while in this case the division of the resisting muscles and of the newly-formed joint not only rendered the reduction possible, but at the same time diminished its after consequences. The limb is now again restored to perfect utility.

The professor adds that he had lately occasion to reduce a luxation of the foot backwards of upwards of a year's standing, by dividing the tendo achillis, which forcibly drew the heel upwards. This limb also became useful again.—*Ib.*, *from Medicinische Zeitung*. Dec. 18, 1839.

---

*On the Cure of Congenital Squinting by Division of the Internal Straight Muscle of the Eye.* By Professor DIEFFENBACH, of Berlin.—[The following cases are masterpieces from the hand of a master. The operation is beautiful in its simplicity, and the result delightful to contemplate. Who shall set bounds to the progress of surgery? These operations are the first of the kind ever performed on the living subject; but Stromeyer has the merit of having suggested the operation, and he performed it on the dead body, with a direct view to proving its practicability on the living.]

*Case* 1.—The subject of this operation was a child seven years old, whose eye was drawn far into the inner angle of the eyelids, so as to produce considerable disfigurement. The operation was performed in the following manner:—The head of the child was held against the chest of one assistant, while another with two hooks kept the eyelids widely apart. The operator then passed a third hook, which he gave to a third assistant to hold, through the conjunctiva, and to some depth in the subjacent cellular tissue at the internal canthus. He next fixed a fine double hook in the sclerotica at the inner angle, and taking it in his left hand drew the eye outwards. Then cutting into the conjunctiva close to the ball where it is continued from it to the internal canthus, and penetrating more deeply by separating the cellular tissue by the side of the sclerotica, he divided the internal rectus muscle close to its insertion with a fine pair of scissors. The eye was immediately drawn outwards by the external rectus, as if it had received an electric shock; and in another instant became straight, so that there was no difference perceptible between its direction and that of the other eye.

The hæmorrhage during the operation was but slight, though sufficient to impede it. The after-treatment consisted of cold lotions; no inflammation ensued, and within eight days the cure was completed.

*Case* 2.—Carl Gerhard, æt. ten, affected with squint since his fourth year. His parents wishing him to become a printer, were anxious to have this defect removed, as it interfered with composing. The right eye was so completely drawn into the inner angle, that on a first view the point of junction of the iris and sclerotica formed the centre of the anterior surface of the eyeball. By an effort the eye could be drawn from the canthus and placed straight, but could not be turned at all outwards. The operation was performed as in the last case, the conjunctiva being cut through, and the sclerotica laid bare to the extent of four lines, in order to bring the muscle into view, which was cut with a curved scissors as before. The squint was gone; the eyeball, when at rest, stood nearly straight, or rather a little turned outwards; and could be turned more readily by the patient's efforts in this direction than inwards. All the other movements of the eye were free. The bleeding was here much less than in the former case, and caused no interruption. The sudden turning of the eyeball outwards, observed in the first case, did not take place here.

The boy felt quite well on the following day. He could separate the eyelids without difficulty. The conjunctiva in the inner angle of the eye was red. The eye was nearly straight, only turned a little more outwards than the other. In eight days the cure was complete, and the eye quite straight.

*Case* 3.—Albert Victor, æt. fifteen, affected with strabismus of the left eye since his earliest infancy. The eyeball was turned deeply into the inner angle; but by an effort of the will it could be turned straight, but on this effort being relaxed, it instantly returned to the former position. The operation was performed precisely in the same manner, it being only here specified that the external incision in the conjunctiva was semilunar, and that the muscle was cut by introducing the pointed blade of the scissors beneath it. As soon as the hook that held the eye was removed, the ball turned at first outwards, but in a moment returned to the straight position. The edges of the wound did not gape, so that the external incision was barely perceptible. The eye was covered with a cold poultice, and the patient subjected to the antiphlogistic regimen. In eight days the cure was complete, and the squint entirely gone.—*Ib.*, *from Medicinische Zeitung*. Nov. 13, 1839, and Feb. 5 and 12, 1840.

---

*Notice of a New Monstrosity*; *A portion of a Fœtus living upon the Testicle.* By M. VELPEAU.—The case on which I propose to engage the Academy to-day, is one of the most strange that the sciences of observation have yet had to consider; interesting at once to surgery, pathology, anatomy, generation, physiology in general, it appears to be without parallel among known facts. It relates to a living portion of a fœtus fixed in the testicle of an adult, where it seems to have been developed and to have lived since his birth. This is a peculiarity so contrary to what we know, and is at first glance so incomprehensible, that one might be justified in doubting its existence if I did not possess the substantial proof of it in the preparations here presented, and if the patient and the tumour had not been observed by several hundreds of practitioners and students, and if the operation had not been performed in the presence of 500 persons. The case is, in a few words, as follows:—

A young man, named Gallochat, of Esterney, aged 27, of a good constitution, and who had never suffered from any severe disease, was sent, in the middle of January, to M. Andral, who at once passed him over to my division in the Hôpital de la Charité.

On examination, I found that the patient had a tumor, nearly as large as a fist, on the right side of the scrotum. It appeared unconnected with the substance of the testicle; the skin over it presented no analogy to that of the scrotum, and it did not appear to me to belong to any known class of tumors. Although several surgeons thought it might be referred, some to the cancerous tumors, some to the fibrous, and some to the tuberculous class, I did not think it possible to adopt their opinion. Observing, moreover, that its origin dated back to the patient's birth, that it was not perceived at its commencement, that it had never produced any pain, that no pathological process had been set up in it, and that it could be cut, or pricked, or pierced through and through, without causing the least suffering; taking notice also of the aspect of the skin which covered its external surface, of its elasticity, of the indurations which it presented internally, of a tuft of hair which came from a kind of ulcer at its posterior part, of a reddish tubercle at the bottom of another opening anteriorly, and of a glairy or grumous matter which the patient had sometimes dischaged; I came to the idea that it was a *fœtal tumor*, *a product of conception.*

Wishing to obtain exact information on the earliest history of so singular a production, I wrote to M. Senoble, physician at Esternay, who answered me thus:—"At the age of about four months, the mother of Gallochat came to show me her child; he then had a tumor, or merely a swelling of the scrotum, which I found to be only a pneumatocele. Some months afterwards, I found, on examining him again, a small inflamed tumor, which appeared to me to be a slight phlegmon, and which yielded to simple emollient local applications. I heard no more of him till at the end of three or four years, when I learned that the child's tumor still continued enlarging." Now although these details were very incomplete, they yet strengthened me in my first opinion; which seemed so singular to those to whom I mentioned it, that I alone held it. I therefore planned the removal of the tumor without taking away the testicle, intending to perform a kind of *Cæsarean operation on the man.* The details of the proceeding belong entirely to surgery, and need not now occupy me; it may be sufficient to state that its results were satisfactory.

The examination of the tumor has enabled me to detect nearly all the anatomical elements of the body of a mammal. Thus, its external layer is evidently cutaneous; the greater part of its substance is a mixture of lamellæ and fibres which give the idea of the cellular, adipose, musular, and fibrous tissues. In its interior, we found two small cysts filled with matter like albumen or the vitreous humour of the eye; another cyst, as large as a partridge's egg, contained a greenish yellow and semi-liquid matter like meconium; in a fourth sac there was a grumous substance, of a dirty-yellow colour, concrete, and surrounded with hair. The substance from this last sac, when analyzed and examined with the microscope, presented all the characters of sebaceous matter and scales of epidermis. The hairs did not appear to have any bulbs at their bases. The tuft of hair which was seen externally, protruded from one of these cysts—from that which was filled with greenish matter; and the opening in it had some analogy with an anus.

Lastly, in the midst of all these elements, we found numerous portions of the skeleton perfectly organized, evidently belonging (as any one may convince himself by examining the preparation) to true bones, and not to accidental productions. These bones, which were every where enveloped by a sort of periosteum, and of which the several pieces were moveable upon each other, and had distinct articulations, may be divided into three sets. The first group is essentially composed of three pieces, in which I thought I could recognize the clavicle, the scapula, and a part of the humerus. The second group, much larger than the preceeding, appears to belong to the pelvis, or perhaps to the base of the skull; the body of the sphenoid, or else the sacrum, forms the central portion. Lastly, the third series seems to comprehend portions of vertebræ and fragments of undetermined bones.

Whatever be the name that the different portions I have pointed out may deserve, certain it is that they belong to a product of conception, and to a fœtus already far advanced in its developement. They are before the Academy, and the correctness of the fact is absolutely incontrovertible. In the monstrosity by inclusion, as it is called, which has been described by Dupuytren, Geoffroy, and Ollivier, one of the fœtuses absorbed by the other has always appeared surrounded by a cyst, and in the condition of a foreign body in the tissues of the fœtus which has continued alive. In the cases related by Saint-Donat, Prochaska, and others, of the debris of fœtus contained in the scrotum, there have always been encysted tumors, necrosed bones, and organized parts destroyed by suppuration and in a state of decomposition. In this subject, on the contrary, every thing has continued to live. The anormal tumor had its own proper colour, consistence, and sensibility, entirely independent of the individual who supported it; a clear well-defined line separated the integuments of its skin from the scrotum. I pinched it with all possible force; I pricked it with various instruments: the young man himself several times ran a knife into it, without feeling the least painful sensations; and yet all the wounds that were made in it bled abundantly, inflamed, and cicatrized, like those of any other part of the body, and nothing indicated in it the least diseased condition. The substances, and all the elements that were found in it, gave the idea of normal tissues or products, and we were quite unable to discover the existence of the least drop of pus, or of any carious or necrosed bone, any altered cartilage, or the least fungous production.

When, on the other hand, one observes that the tumor was as large as a fist—that the surgeon who saw the child when four months old scarcely took notice of it, and that he took it at first for a pneumatocele, and then for a little phlegmon, which terminated by resolution—it is difficult to believe that its volume was as considerable at the birth of the patient as it was the time when I first saw it. Such a mass in an infant would certainly have attracted great attention both from the physician and the family. We must remember, moreover, that, according to M. Senoble's statement, the tumor continued to grow at least up to the age of six or seven years, and that the young man, who says that it has always had the same appearance, can scarcely charge his memory so far back as that time of his life: we must therefore conclude that the portions of the fœtus which I have described have lived and been developed simultaneously with the individual who bore them, and that there were thus two beings united to one another.

Now how could this take place? Did a part of the fœtus, the remainder of which has disappeared, become attached, during intra-uterine life, to the scrotum, in such a manner as to remain there in the form of a graft?—or can this be the remains of a fœtus which at first passed into the abdomen of another, and then descended by the tunica vaginalis, and has at last worn away from within outwards the envelopes of the scrotum?—or, lastly, have we here a creation, the unaided product, of the testicle? But I desist; these are delicate questions in high physiology and in transcendental anatomy, which I am neither able nor willing to broach till the preparations which suggest them have been submitted to the judgment of the Academy.—*London, from Paris Medical Gazette, Feb.* 15, 1840.

---

*Case of Dislocation of the Elbow, in which the Radius and Ulna were thrown forwards, with Fracture of the Ulna.* By M. Richet.—A man fell from a high scaffold, as it was supposed, on his back and the palm of the left hand, and was admitted into the Hôpital St. Louis, in a state of partial collapse.

On examining the bend of the elbow, a hard oblong tumour was seen a finger's breadth above the condyles of the humerus, elevating the biceps and brachialis internus, and rendering the brachial artery comparatively superficial. This tumour was moveable on bending and extending the fore-arm, and rolled under the finger towards the radial side during supination and extension, giving rise to an indistinct crepitation. The olecranon was prominent, moveable transversely, but maintained its natural position. On tracing the edge of the ulna downwards, a flesh-wound was seen two fingers' breadth below the olecranon, through which the bone protruded. The condyles of the humerus, though preserving their natural relations with the posterior part of the ulna, projected considerably, so as to stretch the skin.

The dislocation being immediately recognised as that of the radius and ulna forwards, with fracture of the ulna, was easily reduced by extending the fore-arm, and then suddenly and forcibly bending it, at the same time forcing the upper extremities of the radius and ulna downwards and backwards. All deformity quickly vanished, the bones having regained their natural position, when, in consequence of extension having been remitted, an involuntary contraction reproduced the dislocation with the utmost ease. It was again reduced, and kept so, by means of a cushion, and bandage surrounding both forearm and arm—the forearm being bent at a right angle with the arm, and the cushion filling up the angle and keeping the bones in place—at the same time a splint was applied along the inner side of the joint. The patient died three hours after, from the other injuries received.

On examination, it was found that the triceps was firmly attached to the whole of the posterior fragment of the ulna, as were also the extensor carpi ulnaris and anconæus, thus accounting for the natural position of the olecranon. A longitudinal fracture was seen, somewhat oblique, from above downwards, and from before backwards, running along the middle of the sigmoid cavity to the external border of the ulna, and thence backwards and inwards to a point a finger's breadth below the olecranon, which was the part that had protruded.

The ulna was thus divided into two fragments, the larger formed by the body, surmounted by the coronoid process; the smaller and posterior by the olecranon, and an inch and a half of the ulna below it, ending in a point, which constituted the protrusion. The radius and ulna were seen on the front of the humerus, half an inch above its condyles. The capsular ligament was torn throughout, which explained the easy reduction and reproduction of the luxation.

[This case is worthy of remark, since it proves the possibility of a dislocation of the elbow forwards *without* fracture of the olecranon, contrary to the assertion of Sir A. Cooper and others.]—*Archives Générales de Médecine.*

